# Spleen and Bruton’s tyrosine kinase inhibitors for the management of adult immune thrombocytopenia: A systematic review and meta-analysis of randomized controlled trials

**DOI:** 10.46989/001c.154588

**Published:** 2025-12-24

**Authors:** Akhil D. Vatvani, Timotius I. Hariyanto, Alejandrina C. Ramirez, Nehad Shabarek

**Affiliations:** 1 Department of Internal Medicine, NYC Health + Hospitals/Lincoln, Bronx, New York, USA; 2 Faculty of Medicine, Pelita Harapan University, Karawaci, Tangerang, 15811, Indonesia

**Keywords:** tyrosine kinase inhibitors, spleen tyrosine kinase, bruton’s tyrosine kinase, immune thrombocytopenia, meta-analysis

## Abstract

Spleen tyrosine kinase (SYK) and Bruton’s tyrosine kinase (BTK) inhibitors have emerged as promising targeted therapies for adult immune thrombocytopenia (ITP). However, a comprehensive synthesis of their benefits compared to placebo has not been previously conducted. This study aims to analyze the efficacy and safety of SYK and BTK inhibitors in comparison to placebo for the treatment of adult patients with ITP. A systematic search was performed across four major databases (Medline, Europe PMC, Scopus, and ClinicalTrials.gov) for randomized controlled trials (RCTs) evaluating SYK and/or BTK inhibitors versus placebo in adults with ITP. We utilized random-effects models to evaluate the risk ratio (RR) and mean difference (MD) for the occurrence of outcomes. Five RCTs across four publications were included. Both SYK and BTK inhibitors significantly improved overall platelet response (RR 3.95; 95%CI: 2.68 – 5.81, p<0.00001) and durable response rates (RR 12.50; 95%CI: 3.99 – 39.18, p<0.0001) compared to placebo. Treatment also resulted in shorter time to response and reduced need for rescue interventions. Although total AEs and rash were more common in the intervention group, these were predominantly grade 1–2 and did not lead to increased discontinuation or serious AE rates. No significant differences were observed in the specific events such as gastrointestinal symptoms, cytopenias, liver enzyme elevations, infections, or hypertension. SYK and BTK inhibitors demonstrate superior efficacy over placebo in improving platelet outcomes in adult ITP without compromising safety. These findings support their role as effective treatment options, especially for patients unresponsive to other therapies.

## Introduction

Immune thrombocytopenia (ITP) is an acquired autoimmune disorder in which pathogenic autoantibodies and cytotoxic T cells contribute to the destruction and impaired production of platelets, leading to thrombocytopenia and an increased risk of bleeding.^1^ In adults, ITP often follows a chronic or relapsing course, with many patients requiring long-term treatment beyond first-line therapies such as corticosteroids or intravenous immunoglobulin (IVIG).^2,3^ However, conventional second-line treatments—including splenectomy, rituximab, and thrombopoietin receptor agonists—are associated with variable efficacy, risk of relapse, or long-term safety concerns, underscoring the need for novel, targeted therapies.^2,3^

In recent years, tyrosine kinase inhibitors (TKIs) have emerged as promising agents for modulating the underlying immune mechanisms of ITP.^4,5^ Among them, spleen tyrosine kinase (SYK) inhibitors (e.g., fostamatinib) and Bruton’s tyrosine kinase (BTK) inhibitors (e.g., rilzabrutinib) have shown particular potential.^4,5^ SYK plays a central role in Fc receptor-mediated phagocytosis of platelets by macrophages, while BTK is critical for B-cell receptor signaling and autoantibody production.^4,5^ Inhibiting these kinases may disrupt key pathways in the pathogenesis of ITP, thereby reducing platelet destruction and restoring platelet counts.^4,5^

Preliminary clinical trials and observational studies have demonstrated encouraging results with both SYK and BTK inhibitors in adult ITP, including acceptable safety profiles and clinically meaningful platelet responses in patients refractory to standard treatments.^6,7^ However, the existing evidence is limited by small sample sizes, heterogeneous study designs, and a lack of head-to-head comparisons or long-term follow-up data.^6,7^ Moreover, while individual studies have reported varying efficacy and safety outcomes, no comprehensive synthesis has been performed to date that systematically compares and evaluates both classes of TKIs.

Given the clinical relevance of these agents and the expanding body of early-phase and late-phase research, a meta-analysis is warranted to assess their overall efficacy and safety in adult patients with ITP. Such an analysis would enable more robust estimates of treatment response rates (e.g., durable response, overall response), rates of bleeding reduction, adverse events, and treatment discontinuation. By consolidating available evidence, this meta-analysis aims to support clinicians in evidence-based decision-making, by analyzing the efficacy and safety of SYK and BTK inhibitors for adult patients with ITP.

## Materials and Methods

### Eligibility criteria

This systematic review and meta-analysis were conducted in accordance with the PRISMA guidelines.^8^ The study protocol was prospectively registered with PROSPERO (CRD420251082446). Studies were deemed eligible for inclusion based on criteria structured around the PICOS framework, detailed as follows:

1. Population (P): Adults (≥18 years) diagnosed with immune thrombocytopenia (ITP), either persistent or chronic, who have documented intolerance, inadequate response, or any contraindication to at least one standard ITP treatment.
2. Intervention (I): Treatment with SYK inhibitors (e.g., fostamatinib) or BTK inhibitors (e.g., rilzabrutinib).
3. Control (C): Placebo or best supportive care used in clinical trials.
4. Outcome (O):
  - Efficacy outcomes: durable platelet response, overall platelet response, mean platelet count, number of rescue treatments, and time to response;
  - Safety outcomes: total adverse events (AEs), grade ≥ AEs, serious AEs, treatment discontinuation due to AEs, liver enzyme elevation, nausea, vomiting, diarrhea, abdominal pain, anemia, rash, neutropenia, upper respiratory infection, and hypertension.
5. Study Design (S): phase 3 randomized controlled trials (RCTs).

Concurrently, the subsequent studies were omitted: (1) Studies focusing exclusively on pediatric populations with ITP; (2) Research involving participants with newly-diagnosed ITP that had not undergone any treatment before; (3) Research utilizing animals or cell-based models as subjects of inquiry; (4) Investigation lacking comparison group; (5) Phase 1 or 2 RCTs; (6) Protocol, editorial correspondence, case report, case series, cohort, case-control, cross-sectional studies, and non-primary research; (7) Scholarly publications that were not obtainable in their entirety or research that had not yet completed the publication procedure.

### Search Strategy and Study Selection

We performed a comprehensive search for literature in scientific search engines including Scopus, Medline, ClinicalTrials.gov, and Europe PMC until June 30th, 2025. English language articles were favored without limitations on the publication timeframe. The search employed a combination of MESH and non-MESH phrases, as detailed below: “(tyrosine kinase inhibitors OR spleen tyrosine kinase inhibitors OR SYK inhibitors OR Bruton tyrosine kinase inhibitors OR BTK inhibitors OR fostamatinib OR sovleplenib OR rilzabrutinib OR zanubrutinib OR ibrutinib OR acalabrutinib OR orelabrutinib) AND (immune thrombocytopenia OR immune thrombocytopenic purpura OR idiopathic thrombocytopenic purpura OR ITP) AND (trial OR clinical trial OR randomized trial OR randomized controlled trial)”. All search results were imported into reference management software (Zotero) and duplicates were subsequently removed. Any primary research publications cited in the systematic reviews or meta-analyses, which were not initially identified during the search procedure, but satisfied the predetermined inclusion and exclusion criteria, were be integrated into the study. Two reviewers independently screened titles and abstracts to identify potentially eligible studies. Full-text articles of shortlisted studies were retrieved and reviewed for final inclusion. Disagreements between reviewers were resolved through discussion or by consulting a third reviewer.

**Figure 1.**
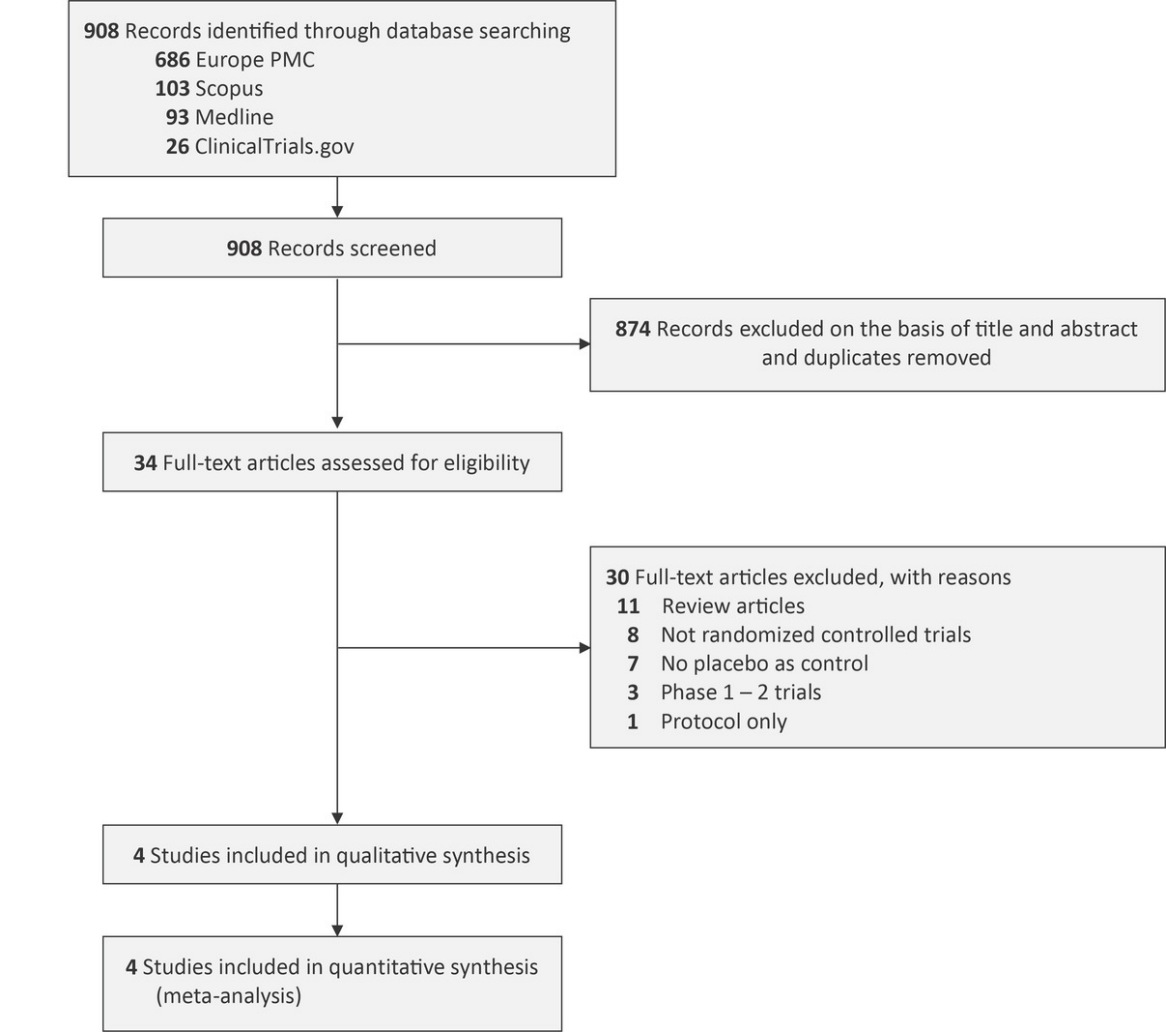
PRISMA diagram of the detailed process of selection of studies for inclusion in the systematic review and meta-analysis.

### Data Extraction

A standardized data extraction form was used by two independent reviewers to extract the following information from each included study: study characteristics (author(s), publication year, study design, sample size); population characteristics (age, gender, ITP duration, prior treatments); intervention details (type of TKI, dosage, treatment duration), as well as outcomes of interest.

The outcomes of interest in this study were divided as the efficacy and safety outcomes. The definition for each efficacy outcome was explained in the following statements. Durable platelet response rate was defined as the number of participants achieving a platelet count ≥50 × 10⁹/L during no fewer than four out of six (at least two-thirds) predetermined assessment points in last 10 or 12 weeks of follow-up period without any rescue therapy.^9^ Overall platelet response rate was defined as the number of individuals who recorded at least one instance of a platelet count ≥50 × 10⁹/L at any point throughout the treatment or follow-up phases.^9^ Time to response was measured as the interval between the commencement of therapy and the first observed platelet count of ≥50 × 10⁹/L.^9^ Mean platelet count was described as the average numbers of platelet in × 10⁹/L, measured at the last day of follow-up period. Rescue treatment of ITP was defined as the use of medications (e.g., corticosteroids) that focus on rapidly increasing platelet counts to manage severe bleeding or the risk of it.^9^ Short corticosteroid bursts used for minor fluctuations or transient platelet drops were not considered rescue therapy, unless they met predefined criteria for treatment failure or required prolonged administration.^9^

Concurrently, the safety outcomes mainly encompass the incidence of AEs. AEs were classified on the basis of severity: 1) grade 1 or mild AEs (generally not bothersome, and possibly not requiring intervention); 2) grade 2 or moderate AEs (possibly causing some discomfort or interference with activities, and requiring minimal intervention); and 3) grade 3 or severe AEs (significantly interfering with a person’s ability to perform basic self-care activities and requiring medical interventions).^10^ For the purpose of analysis, we divided the severity of each specific type of AE into only two categories, namely mild-to-moderate (grade 1 – 2) and severe (grade ≥3).

### Risk of Bias Assessment

To evaluate methodological rigor of the RCTs included in this review, the Cochrane Risk of Bias 2.0 (RoB 2) instrument was employed.^11^ This tool examines five distinct areas where bias may occur: (a) the process of random sequence generation and allocation; (b) inconsistencies or deviations from the assigned interventions; (c) absence or loss of outcome data; (d) the method used to assess outcomes; and (e) selective outcome reporting.^11^ Each domain was categorized as presenting a “low risk,” “some concerns,” or “high risk” of bias.^11^ Two reviewers independently conducted the risk of bias evaluations, and any differences in judgment were settled through consultation with a third reviewer.

### Statistical Analysis

For continuous variable outcomes, the mean difference (MD) and corresponding 95% confidence intervals (CI) were estimated using the Inverse Variance method. In contrast, dichotomous variable outcomes were analyzed through the Mantel-Haenszel approach to determine risk ratios (RR) along with their 95% CIs. Given the heterogeneity in TKI regimens and participant profiles across studies, substantial variability was anticipated. To address this, random-effect models were applied. Between-study heterogeneity was quantified using the I² statistic, with values exceeding 50% interpreted as indicating considerable inconsistency.^12^ For the purpose of pooling data in the meta-analysis, outcomes originally reported as medians with interquartile or minimum-to-maximum ranges were transformed into means and standard deviations under the assumptions of approximate normality, symmetrical distribution around the median, sufficient sample size, and absence of extreme outliers. This transformation was performed using the statistical methods proposed by Wan X et al.^13^ and Luo D et al.^14^ In cases where the meta-analysis included more than 10 studies, potential publication bias was evaluated using funnel plot visualization. All statistical computations were carried out using Review Manager (RevMan) version 5.4, developed by the Cochrane Collaboration.

## Results

### Study Selection and Characteristics

An extensive database exploration across four platforms, guided by predefined search terms, initially retrieved 908 records. After eliminating duplicates, preliminary screening based on titles and abstracts led to the removal of 874 entries. The remaining 34 articles underwent full-text evaluation. Following the application of exclusion parameters, 30 studies were dismissed: 11 were categorized as reviews, 8 lacked randomization, 7 did not use a placebo comparator, 3 were early-phase trials (phases 1 or 2), and 1 was identified as a protocol-only publication. As a result, five RCTs derived from four publications^15–18^ were deemed eligible and subsequently included in the meta-analysis. Four out of five included RCTs employing double-blind design, while the remaining RCT used open-label design. The type of TKI administered among the participants of included RCTs was relatively varied: fostamatinib 100 – 150 mg twice daily in three RCTs, sovleplenib 300 mg once daily in one RCT, and rilzabrutinib 400 mg twice daily in one RCT. All of these interventions were given for a total of 24 weeks. The sample sizes in the TKI group ranged from 22 to 133 patients, while in the placebo group it varied from 12 to 69 patients. Prior ITP treatments most commonly prescribed to the participants were corticosteroids, intravenous immunoglobulin (IVIG), thrombopoietin receptor agonist (TPO-RA), and immunosuppressants. Table 1 presents a detailed summary of the features of each study incorporated in this analysis.

**Table 1.** Baseline characteristics of the included studies

Study	Design	Intervention	Follow-up (weeks)	Study arms	Sample size	Age (years)	Male (%)	ITP duration (years)	Prior splenectomy (%)	Prior ITP therapy (4 most prevalent)
Bussel J et al.^^15^^ 2018 (FIT1)	Double-blind RCT	Fostamatinib 100 – 150 mg, twice daily (PO)	24	TKI	51	56.4 ± 15.1	41%	10.8 ± 11.6	39%	CS: 90%, IVIG: 65%, TPO-RA: 53%, rituximab: 51%
				Placebo	25	55.5 ± 12.9	32%	10 ± 11.3	40%	CS: 100%, IVIG: 68%, TPO-RA: 60%, rituximab: 44%
Bussel J et al.^^15^^ 2018 (FIT2)	Double-blind RCT	Fostamatinib 100 – 150 mg, twice daily (PO)	24	TKI	50	50.2 ± 13.5	38%	11.6 ± 11.1	28%	CS: 96%, IS: 44%, TPO-RA: 40%, IVIG: 38%
				Placebo	24	49.7 ± 14.8	46%	11.9 ± 7.2	38%	CS: 92%, IVIG: 42%, TPO-RA: 42%, IS: 42%
Hu Y et al.^^16^^ 2024	Double-blind RCT	Sovleplenib 300 mg, once daily (PO)	24	TKI	126	43.6 ± 10.4	31%	8.6 ± 6.7	4%	CS: 96%, TCM: 59%, TPO-RA: 52%, IVIG: 50%
				Placebo	62	42.2 ± 10.9	40%	9.8 ± 8.6	5%	CS: 97%, TCM: 60%, TPO-RA: 52%, IVIG: 47%
Kuter DJ et al.^^17^^ 2025	Open-label RCT	Rilzabrutinib 400 mg, twice daily (PO)	24	TKI	133	47.1 ± 11.9	41%	9.7 ± 9.9	28%	CS: 95%, TPO-RA: 66%, IVIG: 54%, IS: 50%
				Placebo	69	46.4 ± 12.6	29%	7.8 ± 7.5	28%	CS: 96%, TPO-RA: 74%, IS: 61%, IVIG: 58%
Kuwana M et al.^^18^^ 2023	Double-blind RCT	Fostamatinib 100 – 150 mg, twice daily (PO)	24	TKI	22	58.7 ± 14.6	18%	14.5 ± 10.4	23%	CS: 95%, TPO-RA: 50%, IVIG: 36%, IS: 14%
				Placebo	12	59.9 ± 13.7	33%	14.8 ± 11.3	17%	CS: 100%, TPO-RA: 58%, IVIG: 33%, rituximab: 33%

CS = corticosteroids; IS = immunosuppressants; IVIG = intravenous immunoglobulin; ITP = immune thrombocytopenia; PO = per os (oral administration); RCT = randomized controlled trials; TCM = traditional chinese medicine; TKI = tyrosine kinase inhibitors; TPO-RA = thrombopoietin receptor agonist

### Quality of Study Assessment

The risk of bias in the included RCTs was assessed using the RoB 2.0 framework. Based on the evaluation across five domains, all trials were determined to exhibit a “low risk” of bias (Table 2).

**Table 2.**
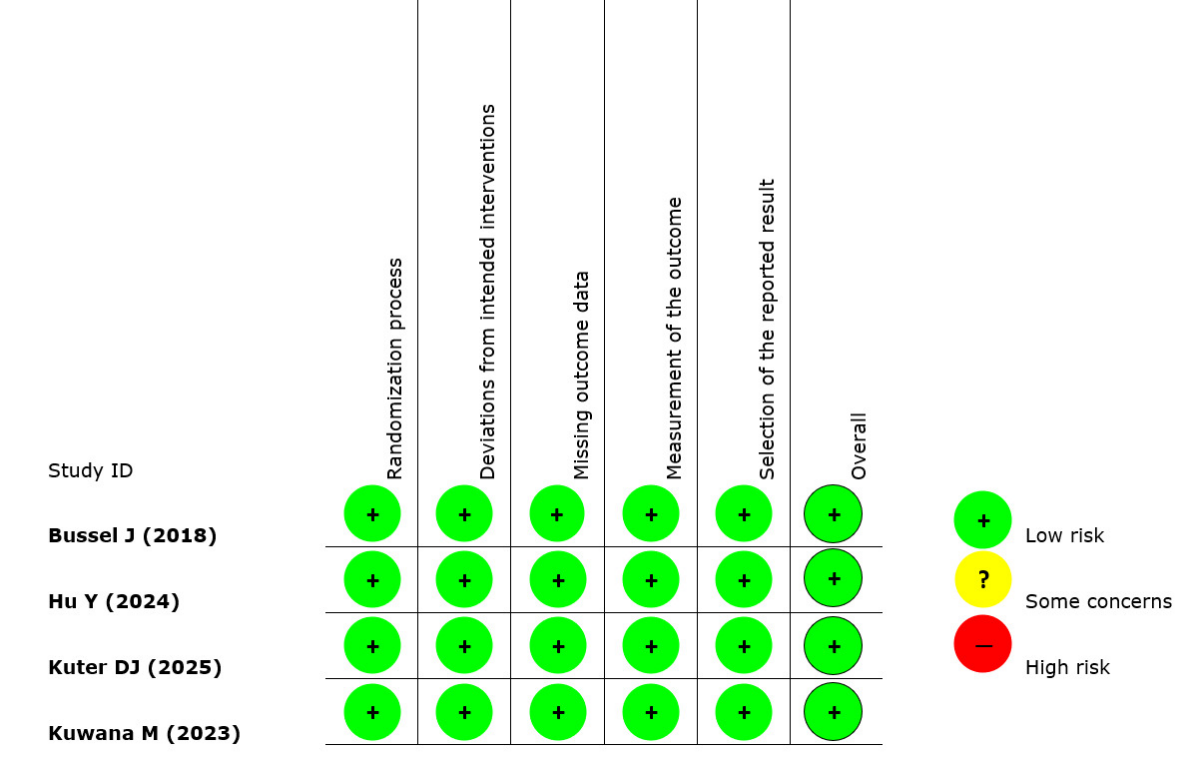
Risk of bias assessment using RoB v2 tool from Cochrane Collaborations

### Efficacy Outcomes

#### Durable Platelet Response

**Figure 2.**
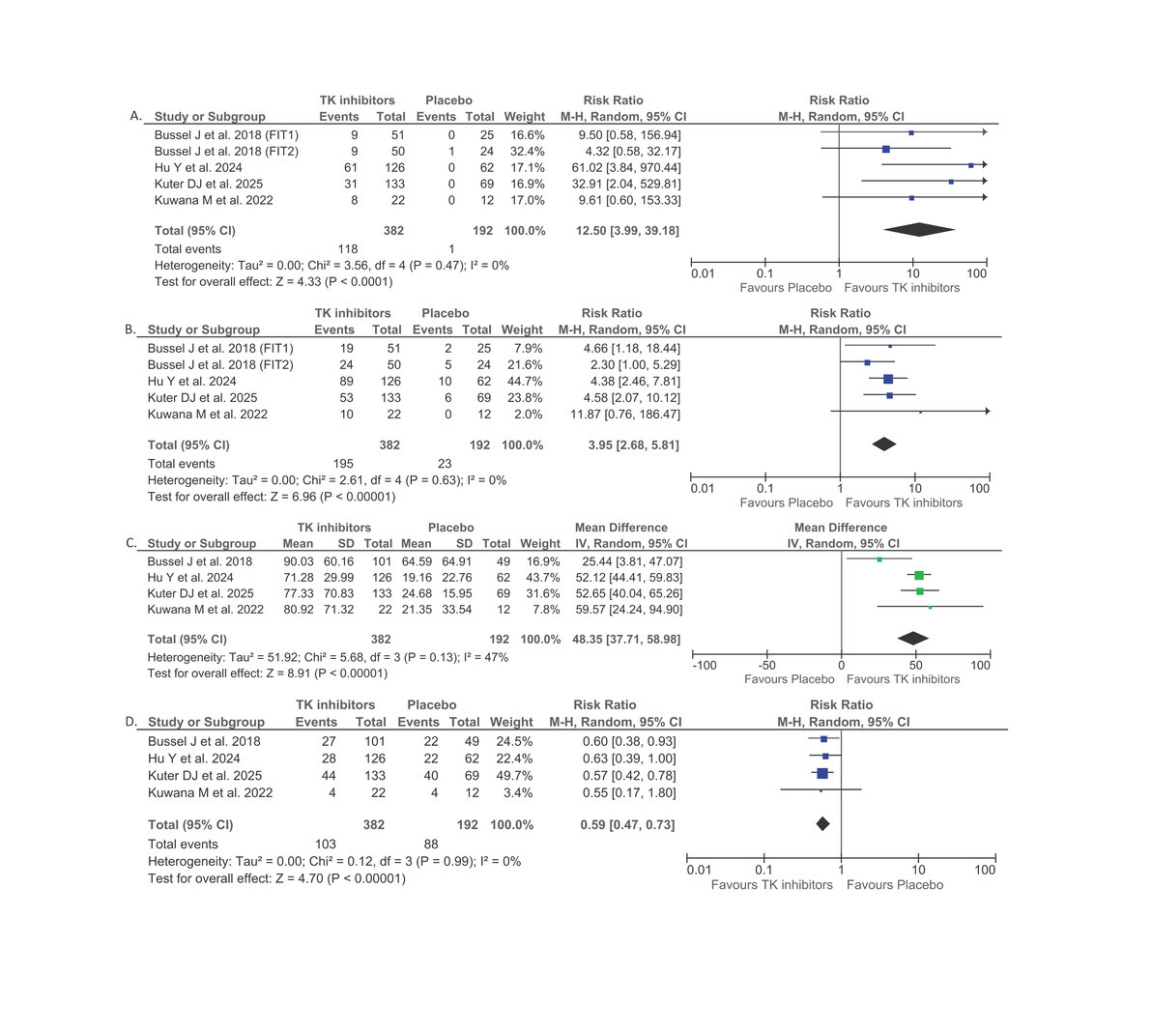
Forest plot that shows the comparison between spleen tyrosine kinase (SYK) or Bruton’s tyrosine kinase (BTK) inhibitors and placebo among adult immune thrombocytopenia (ITP) patients in relation to durable platelet response (A), overall platelet response (B), mean platelet count (C), and the need for rescue treatments (D) outcomes.

A total of 5 RCTs from 4 publications (n = 574) provided data on the durable platelet response outcome. This outcome was achieved in 118 of 382 patients (30.9%) receiving SYK and/or BTK inhibitors compared with only 1 of 192 patients (0.5%) in the placebo group. The pooled analysis of these RCTs indicated that adult ITP patients who were treated with SYK and/or BTK inhibitors exhibited higher likelihood of achieving durable platelet response compared to those who only received placebo (RR 12.50; 95%CI: 3.99 – 39.18, p<0.0001, I^2^ = 0%, random-effect model) (Figure 2A) (Table 3). The subgroup analysis demonstrated that BTK inhibitors were associated with a higher RR of achieving a durable platelet response (RR 32.91; 95%CI: 2.04 – 529.81, p=0.01, random-effect model) compared to SYK inhibitors (RR 10.30; 95%CI: 2.91 – 36.54, p=0.0003, I^2^ = 2%, random-effect model) (Table 3).

**Table 3.** Summary of meta-analysis results for each outcome of interest

**Outcome**	**Patients analyzed**		**Outcome (95% CI)**	**p-value**	**I^2^ (%)**
	**TK inhibitors**	**Placebo**			
**Efficacy Outcomes**					
Durable response rate Overall BTK inhibitors SYK inhibitors	382 133 249	192 69 123	RR: 12.50 (3.99 – 39.18) RR: 32.91 (2.04 – 529.81) RR: 10.30 (2.91 – 36.54)	<0.0001 0.01 0.0003	0 - 2
Overall response rate Overall BTK inhibitors SYK inhibitors	382 133 249	192 69 123	RR: 3.95 (2.68 – 5.81) RR: 4.58 (2.07 – 10.12) RR: 3.77 (2.42 – 5.87)	<0.00001 0.0002 <0.00001	0 - 0
Mean platelet count Overall BTK inhibitors SYK inhibitors	382 133 249	192 69 123	MD: 48.35 (37.71, 58.98) MD: 52.65 (40.04, 65.26) MD: 45.05 (25.98, 64.13)	<0.00001 <0.00001 <0.00001	47 - 64
Rescue treatments Overall BTK inhibitors SYK inhibitors	382 133 249	192 69 123	RR: 0.59 (0.47 – 0.73) RR: 0.57 (0.42 – 0.78) RR: 0.61 (0.44 – 0.83)	<0.00001 0.0005 0.002	0 - 0
**Safety Outcomes**					
Total AEs Overall BTK inhibitors SYK inhibitors	383 133 250	191 69 122	RR: 1.14 (1.06 – 1.24) RR: 1.11 (0.95 – 1.29) RR: 1.16 (1.06 – 1.26)	0.0006 0.20 0.001	0 - 0
Grade ≥3 AEs Overall BTK inhibitors SYK inhibitors	383 133 250	191 69 122	RR: 0.77 (0.52 – 1.14) RR: 0.78 (0.37 – 1.64) RR: 0.77 (0.42 – 1.41)	0.19 0.51 0.39	12 - 41
Serious AEs Overall BTK inhibitors SYK inhibitors	383 133 250	191 69 122	RR: 0.87 (0.57 – 1.31) RR: 0.78 (0.33 – 1.81) RR: 0.90 (0.56 – 1.44)	0.50 0.56 0.65	0 - 0
AEs lead to treatment withdrawal Overall BTK inhibitors SYK inhibitors	383 133 250	191 69 122	RR: 1.88 (0.75 – 4.73) RR: 8.88 (0.52 – 151.62) RR: 1.57 (0.59 – 4.16)	0.18 0.13 0.36	0 - 0
Liver enzyme elevation Overall Grade 1 – 2 Grade ≥3	383 383 383	191 191 191	RR: 2.17 (0.84 – 5.56) RR: 3.15 (0.80 – 12.51) RR: 1.04 (0.17 – 6.20)	0.11 0.41 0.96	8 31 0
Nausea Overall Grade 1 – 2 Grade ≥3	383 383 383	191 191 191	RR: 1.45 (0.84 – 2.51) RR: 1.71 (0.90 – 3.24) RR: 0.35 (0.05 – 2.21)	0.18 0.10 0.27	23 10 0
Vomiting Overall Grade 1 – 2 Grade ≥3	281 281 281	143 143 143	RR: 1.38 (0.40 – 4.78) RR: 4.24 (0.79 – 22.76) RR: 0.32 (0.04 – 2.60)	0.60 0.09 0.29	0 0 0
Diarrhea Overall Grade 1 – 2 Grade ≥3	257 257 257	129 129 129	RR: 1.95 (0.78 – 4.90) RR: 2.15 (0.66 – 6.96) RR: 1.56 (0.17 – 14.50)	0.15 0.20 0.70	57 79 0
Abdominal pain Overall Grade 1 – 2 Grade ≥3	257 257 257	129 129 129	RR: 1.73 (0.62 – 4.78) RR: 2.36 (0.75 – 7.41) RR: 0.52 (0.05 – 4.87)	0.29 0.14 0.56	0 0 0
Anemia Overall Grade 1 – 2 Grade ≥3	259 259 259	131 131 131	RR: 0.92 (0.32 – 2.62) RR: 1.20 (0.26 – 5.55) RR: 0.48 (0.13 – 1.80)	0.87 0.81 0.27	48 69 0
Rash Overall Grade 1 – 2 Grade ≥3	383 383 383	191 191 191	RR: 2.39 (1.01 – 5.70) RR: 3.37 (1.26 – 8.98) RR: 0.73 (0.12 – 4.59)	0.05 0.02 0.74	0 0 0
Neutropenia Overall Grade 1 – 2 Grade ≥3	383 383 383	191 191 191	RR: 2.84 (0.91 – 8.87) RR: 4.24 (1.00 – 18.01) RR: 1.49 (0.24 – 9.39)	0.07 0.05 0.67	0 0 0
Upper respiratory tract infection Overall Grade 1 – 2 Grade ≥3	383 383 383	191 191 191	RR: 1.42 (0.83 – 2.41) RR: 1.39 (0.59 – 3.26) RR: 0.90 (0.15 – 5.47)	0.19 0.45 0.91	31 53 0
Hypertension Overall Grade 1 – 2 Grade ≥3	250 250 250	122 122 122	RR: 1.38 (0.82 – 2.32) RR: 1.44 (0.83 – 2.50) RR: 1.13 (0.12 – 10.51)	0.23 0.19 0.92	0 0 42

#### Overall Platelet Response

There were 5 RCTs from 4 publications (n = 574) which reported the overall platelet response among all of the ITP patients. This response was observed in 195 of 382 patients (51%) treated with inhibitors versus 23 of 192 (11.9%) in the placebo group. Meta-analysis from these studies showed that treatment utilizing SYK and/or BTK inhibitors was associated with higher risk of obtaining overall platelet response compared to placebo for adult ITP patients (RR 3.95; 95%CI: 2.68 – 5.81, p<0.00001, I^2^ = 0%, random-effect model) (Figure 2B) (Table 3). The subgroup analysis revealed a higher RR for obtaining overall platelet response among BTK inhibitors (RR 4.58; 95%CI: 2.07 – 10.12, p=0.0002, random-effect model) than SYK inhibitors (RR 3.77; 95%CI: 2.42 – 5.87, p<0.00001, I^2^ = 0%, random-effect model) (Table 3).

#### Mean Platelet Count

Data from 4 publications (n = 574) were used to pool the mean platelet count outcome. Meta-analysis from these studies showed higher mean platelet count among adult ITP patients who were given SYK and/or BTK inhibitors than those who only received placebo (Mean Difference 48.35 × 10⁹/L; 95%CI: 37.71 – 58.98, p<0.00001, I^2^ = 47%, random-effect model) (Figure 2C) (Table 3). There was a higher MD among BTK inhibitors (MD 52.65; 95%CI: 40.04 – 65.26, p<0.00001, random-effect model) in comparison to SYK inhibitors (MD 45.05; 95%CI: 25.98 – 64.13, p<0.00001, I^2^ = 64%, random-effect model) (Table 3).

#### Rescue Treatments

There were 4 publications which reported the need for rescue treatments among the adult ITP patients. Meta-analysis from these studies revealed that ITP therapy employing SYK and/or BTK inhibitors was associated with lower rate of rescue treatments administration compared to placebo (RR 0.59; 95%CI: 0.47 – 0.73, p<0.00001, I^2^ = 0%, random-effect model) (Figure 2D) (Table 3). The subgroup analysis revealed a slightly lower RR for the need of rescue treatments among BTK inhibitors (RR 0.57; 95%CI: 0.42 – 0.78, p=0.0005, random-effect model) compared to SYK inhibitors (RR 0.61; 95%CI: 0.44 – 0.83, p=0.002, I^2^ = 0%, random-effect model) (Table 3).

#### Time to First Platelet Response

Three publications^15–17^ reported the time to first platelet response. However, due to insufficient data for calculation, we did not perform meta-analysis on this particular outcome. The study by Hu Y et al.^16^ reported a shorter median time to response among ITP patients who were given sovleplenib 300 mg once daily than those who were given only placebo [8 days (8 – 12) vs. 30 days (24 – 46)]. Kuter DJ et al.^17^ also reported similar findings, where median time to platelet response was 36 days in all patients with rilzabrutinib 400 mg twice daily, but it was never achieved with placebo (p<0.0001). Finally, a study by Bussel J et al.^15^ only reported this outcome in the fostamatinib group: the median duration required to achieve a platelet count of ≥50,000/μL was 15 days among overall responders and 15.5 days among those with stable responses.^15^ At time of response, all individuals—except one who had reduced their dosage to 150 mg once daily—were still receiving the 100 mg twice-daily regimen.^15^

### Safety Outcomes

#### Total AEs

**Figure 3.**
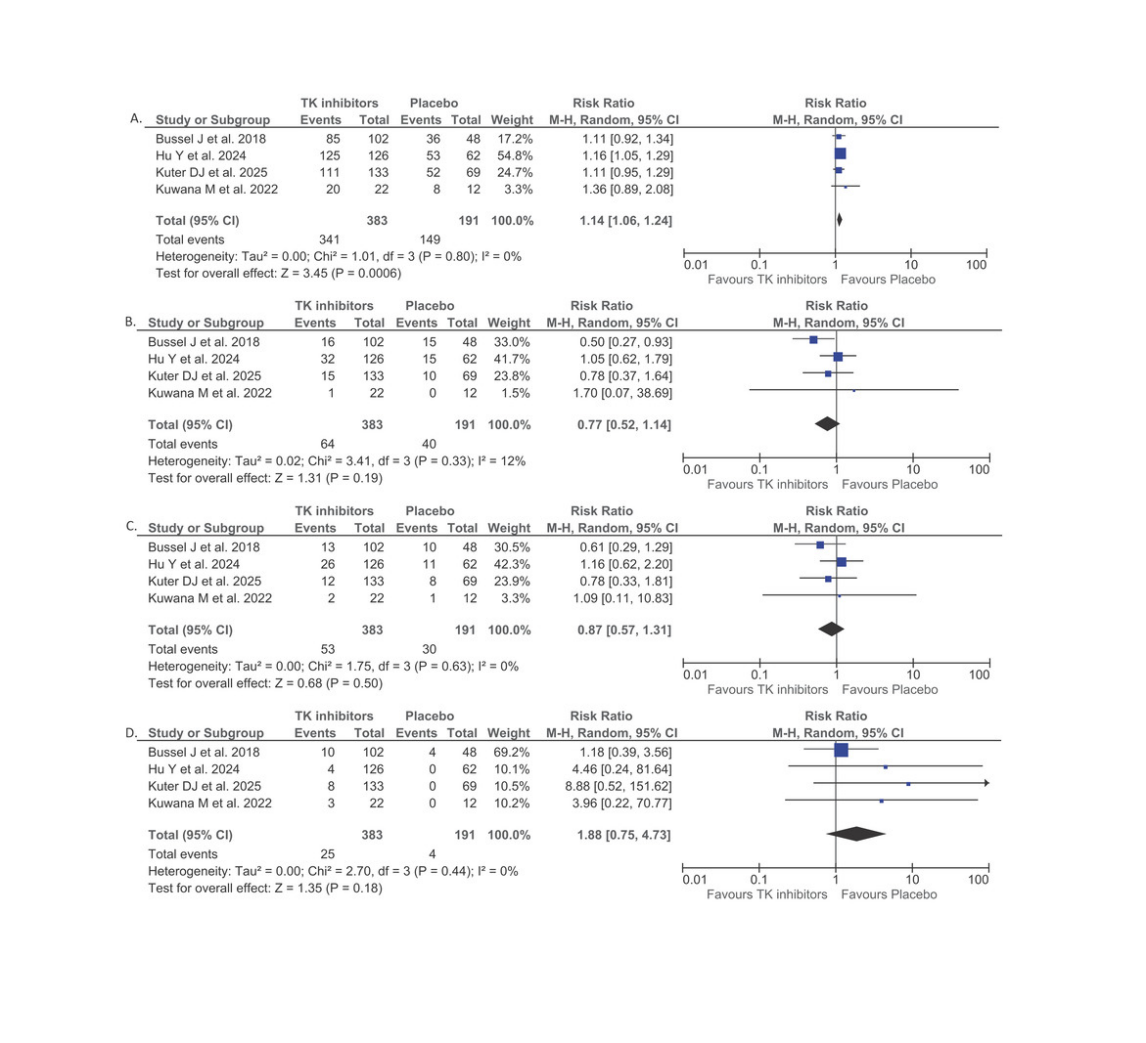
Forest plot that shows the comparison between spleen tyrosine kinase (SYK) or Bruton’s tyrosine kinase (BTK) inhibitors and placebo among adult immune thrombocytopenia (ITP) patients in relation to total adverse events (AEs) (A), grade ≥3 AEs (B), serious AEs (C), and treatment discontinuation due to AEs (D) outcomes.

Four publications (n = 574) reported the total AEs outcome. Pooled analysis from these studies showed higher total AEs among adult ITP patients who received SYK and/or BTK inhibitors than those who received placebo (RR 1.14; 95%CI: 1.06 – 1.24, p=0.0006, I^2^ = 0%, random-effect model) (Figure 3A) (Table 3). The subgroup analysis demonstrated that a significant difference in total AEs was observed only among patients receiving SYK inhibitors (RR 1.16; 95%CI: 1.06 – 1.26, p=0.001, I^2^ = 0%, random-effect model), whereas the results for BTK inhibitors were not significant (RR 1.11; 95%CI: 0.95 – 1.29, p=0.20, random-effect model) (Table 3).

#### Grade ≥3 AEs

Pooled analysis from four publications (n = 574) showed no significant difference in the grade ≥3 AEs incidence between adult ITP patients who were given SYK and/or BTK inhibitors and those who were given placebo (RR 0.77; 95%CI: 0.52 – 1.14, p=0.19, I^2^ = 12%, random-effect model) (Figure 3B) (Table 3). The subgroup analysis revealed almost similar RR between BTK inhibitors (RR 0.78; 95%CI: 0.37 – 1.64, p=0.51, random-effect model) and SYK inhibitors (RR 0.77; 95%CI: 0.42 – 1.41, p=0.39, I^2^ = 41%, random-effect model) (Table 3).

#### Serious AEs

An integrated meta-analysis of four studies (n = 574) demonstrated that the occurrence of serious AEs did not differ significantly between adults with ITP treated with SYK and/or BTK inhibitors and those receiving placebo (OR 0.87; 95%CI: 0.57 – 1.31, p=0.50, I^2^ = 0%, random-effect model) (Figure 3C) (Table 3). The subgroup analysis revealed a lower RR for serious AEs among BTK inhibitors (OR 0.78; 95%CI: 0.33 – 1.81, p=0.56, random-effect model) compared to SYK inhibitors (OR 0.90; 95%CI: 0.56 – 1.44, p=0.65, I^2^ = 0%, random-effect model), but both findings still did not reach statistical significance (Table 3).

#### Treatment discontinuation due to AEs

A pooled analysis of four publications involving 574 participants indicated that the rate of AEs resulting in treatment discontinuation did not significantly differ between adult ITP patients receiving SYK and/or BTK inhibitors and those given placebo (OR 1.88; 95%CI: 0.75 – 4.73, p=0.18, I^2^ = 0%, random-effect model) (Figure 3D) (Table 3). There was a higher RR for treatment discontinuation due to AEs among BTK inhibitors (OR 8.88; 95%CI: 0.52 – 151.62, p=0.13, random-effect model) compared to SYK inhibitors (OR 1.57; 95%CI: 0.59 – 4.16, p=0.36, I^2^ = 0%, random-effect model). However, the difference was not significant (Table 3).

#### Liver Enzyme Elevation

There was no significant difference in the overall liver enzyme elevation incidence between SYK and/or BTK inhibitors and placebo (RR 2.17; 95%CI: 0.84 – 5.56, p=0.11, I^2^ = 8%, random-effect model) (Table 3). The same lack of significant difference was found when divided according to the severity of AEs, as grade 1 – 2 (RR 3.15; 95%CI: 0.80 – 12.51, p=0.41, I2 = 31%, random-effect model) and grade ≥3 liver enzyme elevation (RR 1.04; 95%CI: 0.17 – 6.20, p=0.96, I^2^ = 0%, random-effect model) (Table 3).

#### Nausea

The overall occurrence of nausea did not differ significantly between those treated with SYK and/or BTK inhibitors and those receiving placebo (RR 1.45; 95%CI: 0.84 – 2.51, p=0.18, I^2^ = 23%, random-effect model) (Table 3). Stratification of nausea events by severity revealed no notable differences between groups in either grade 1–2 (RR 1.71; 95%CI: 0.90 – 3.24, p=0.10, I^2^ = 10%, random-effect model) or grade ≥3 occurrences (RR 0.35; 95%CI: 0.05 – 2.21, p=0.27, I^2^ = 0%, random-effect model) (Table 3).

#### Vomiting

Pooled findings from three studies involving 424 adults with ITP indicated that the overall occurrence of vomiting did not differ significantly between those treated with SYK and/or BTK inhibitors and those receiving placebo (RR 1.38; 95%CI: 0.40 – 4.78, p=0.60, I^2^ = 0%, random-effect model) (Table 3). As observed for nausea, stratification of vomiting events by severity revealed no notable differences between groups in either grade 1–2 (RR 4.24; 95%CI: 0.79 – 22.76, p=0.09, I^2^ = 0%, random-effect model) or grade ≥3 occurrences (RR 0.32; 95%CI: 0.04 – 2.60, p=0.29, I^2^ = 0%, random-effect model) (Table 3).

#### Diarrhea

Combined results from three publications including 386 adult ITP patients showed no significant disparity in the overall incidence of diarrhea between individuals administered SYK and/or BTK inhibitors and those given a placebo (RR 1.95; 95%CI: 0.78 – 4.90, p=0.15, I^2^ = 57%, random-effect model) (Table 3). When diarrhea cases were categorized by severity, no meaningful differences were identified between the SYK and/or BTK inhibitors and placebo groups for either grade 1–2 (RR 2.15; 95%CI: 0.66 – 6.96, p=0.20, I^2^ = 79%, random-effect model) or grade ≥3 events (RR 1.56; 95%CI: 0.17 – 14.50, p=0.70, I^2^ = 0%, random-effect model) (Table 3).

#### Abdominal Pain

Combined results from three publications (386 patients) did not show significant difference in the overall incidence of abdominal pain between individuals receiving SYK and/or BTK inhibitors and those given a placebo (RR 1.73; 95%CI: 0.62 – 4.78, p=0.29, I^2^ = 0%, random-effect model) (Table 3).

Likewise, no meaningful differences were identified between the SYK and/or BTK inhibitors and placebo groups for either grade 1–2 (RR 2.36; 95%CI: 0.75 – 7.41, p=0.14, I^2^ = 0%, random-effect model) or grade ≥3 events (RR 0.52; 95%CI: 0.05 – 4.87, p=0.56, I^2^ = 0%, random-effect model) (Table 3).

#### Anemia

Meta-analysis from 3 publications (n = 390) showed no significant difference in the overall anemia incidence between SYK and/or BTK inhibitors and placebo (RR 0.92; 95%CI: 0.32 – 2.62, p=0.87, I^2^ = 48%, random-effect model), regardless of the its severity as grade 1 – 2 (RR 1.20; 95%CI: 0.26 – 5.55, p=0.81, I^2^ = 69%, random-effect model) oor grade ≥3 anemia (RR 0.48; 95%CI: 0.13 – 1.80, p=0.27, I^2^ = 0%, random-effect model) (Table 3).

#### Rash

Meta-analysis from 4 publications (n = 574 patients) showed higher overall incidence of rash in adult ITP patients who received SYK and/or BTK inhibitors when compared to those who received placebo (RR 2.39; 95%CI: 1.01 – 5.70, p=0.05, I^2^ = 0%, random-effect model) (Table 3).

When divided according to the severity of rash, the difference was only significant for grade 1 – 2 (RR 3.37; 95%CI: 1.26 – 8.98, p=0.02, I^2^ = 0%, random-effect model), but not for grade ≥3 rash (RR 0.73; 95%CI: 0.12 – 4.59, p=0.74, I^2^ = 0%, random-effect model) (Table 3).

#### Neutropenia

Pooled findings from four studies (574 patients) indicated that the overall occurrence of neutropenia did not differ significantly between those treated with SYK and/or BTK inhibitors and those receiving placebo (RR 2.84; 95%CI: 0.91 – 8.87, p=0.07, I^2^ = 0%, random-effect model) (Table 3).

Stratification of neutropenia events by severity revealed higher incidence of grade 1 – 2 neutropenia among SYK and/or BTK inhibitors group in comparison to placebo group (RR 4.24; 95%CI: 1.00 – 18.01, p=0.05, I^2^ = 0%, random-effect model). However, no significant difference was observed for grade ≥3 neutropenia (RR 1.49; 95%CI: 0.24 – 9.39, p=0.67, I^2^ = 0%, random-effect model) (Table 3).

#### Upper Respiratory Infection

Combined results from four publications including 574 adult ITP patients showed no significant disparity in the overall incidence of upper respiratory infection between individuals administered SYK and/or BTK inhibitors and those given a placebo (RR 1.42; 95%CI: 0.83 – 2.41, p=0.19, I^2^ = 31%, random-effect model) (Table 3).

When upper respiratory infection cases were categorized by severity, no meaningful differences were identified between the SYK and/or BTK inhibitors and placebo groups for either grade 1–2 (RR 1.39; 95%CI: 0.59 – 3.26, p=0.45, I^2^ = 53%, random-effect model) or grade ≥3 events (RR 0.90; 95%CI: 0.15 – 5.47, p=0.91, I^2^ = 0%, random-effect model) (Table 3).

#### Hypertension

Analysis of three publications including 372 patients showed no significant difference in the overall incidence of hypertension between individuals administered SYK and/or BTK inhibitors and those given a placebo (RR 1.38; 95%CI: 0.82 – 2.32, p=0.23, I^2^ = 0%, random-effect model). The same lack of meaningful differences was identified when comparing hypertension in either grade 1–2 (RR 1.44; 95%CI: 0.83 – 2.50, p=0.19, I^2^ = 0%, random-effect model) or grade ≥3 events (RR 1.13; 95%CI: 0.12 – 10.51, p=0.92, I^2^ = 42%, random-effect model) (Table 3).

### Publication Bias

A funnel plot approach is commonly employed to assess potential publication bias. However, in this analysis, such evaluation was not conducted due to the limited number of eligible studies (fewer than ten), as reliable detection of publication bias generally requires a larger quantity of studies.^19,20^

## Discussion

The results of this systematic review and meta-analysis support the therapeutic potential of TKIs, including both SYK and BTK inhibitors, in managing adult ITP, particularly among individuals who have not responded adequately to or cannot tolerate standard first-line therapies. This is evidenced by favorable outcomes such as increased rates of sustained and overall platelet responses, reduced time to achieve a platelet response, decreased reliance on rescue medications, and elevated mean platelet counts in patients receiving SYK and/or BTK inhibitors compared to those on placebo. The substantial relative risks observed for both overall (RR 3.95) and durable platelet responses (RR 12.5) indicate that treatment with SYK and/or BTK inhibitors confers a marked improvement in hematologic outcomes compared with placebo. In absolute terms, overall platelet response was achieved in 51% patients treated with SYK and/or BTK inhibitors versus 11.9% in the placebo group, while a durable response was observed in 30.9% patients compared with only 0.5% in the placebo arm. These absolute differences suggest a substantial therapeutic gain from a hematologic standpoint. Nevertheless, the clinical meaningfulness of these findings should be interpreted cautiously, as platelet count response represents a surrogate endpoint. Whether these improvements translate into tangible clinical benefits—such as reduced bleeding episodes, sustained remission off treatment, or improved quality of life—remains to be fully established. Although increased durable responses may plausibly reduce bleeding risk and promote longer remission, most available trials had limited follow-up durations, restricting conclusions about long-term efficacy and remission durability. Therefore, while the current evidence supports a notable efficacy advantage for SYK and BTK inhibitors in achieving platelet responses, further long-term and real-world data are required to confirm whether these hematologic benefits consistently translate into meaningful clinical outcomes.

In addition to their clinical efficacy, these agents were generally well tolerated. While the overall incidence of AEs and skin rashes was higher in the treatment group, these were predominantly mild to moderate in severity (grade 1–2) and rarely necessitated intensive management. No meaningful differences were found between groups in the frequency of grade ≥3 AEs, serious AEs, treatment discontinuations due to AEs, or specific safety concerns including elevated liver enzymes, gastrointestinal symptoms (nausea, vomiting, diarrhea, abdominal pain), hematologic abnormalities (anemia, neutropenia), upper respiratory infections, or hypertension. However, it remains important to recognize the established post-marketing safety profile of fostamatinib. Evidence from real-world clinical experience has highlighted hypertension, diarrhea, and hepatotoxicity as the most frequently observed AEs linked to its use. In addition, the limited follow-up duration of the RCTs included in this analysis (approximately 24 weeks) constrains the ability to fully evaluate its long-term safety and to identify uncommon adverse events that may arise with extended treatment.

The observed improvement in platelet response among patients with ITP treated with SYK and BTK inhibitors can be attributed to their targeted interference with key immune signaling pathways involved in the pathogenesis of the disease.^4,7^ ITP is primarily driven by immune-mediated platelet destruction and impaired platelet production, both of which are regulated by dysregulated B-cell and Fc receptor activity.^1^ SYK and BTK inhibitors address these mechanisms directly and effectively.

SYK plays a central role in Fcγ receptor signaling, a pathway responsible for mediating the destruction of antibody-coated platelets by splenic macrophages.^21^ By inhibiting SYK, these agents disrupt the intracellular signaling cascade that leads to phagocytosis of platelets, thereby reducing platelet clearance and allowing circulating platelet counts to recover.^21^ Fostamatinib, a first-in-class SYK inhibitor, has demonstrated this mechanism both in preclinical models and clinical settings, leading to sustained increases in platelet levels in a proportion of patients with chronic ITP.^21,22^ Fostamatinib has already received regulatory approval for the treatment of adult ITP and is supported by accumulating real-world evidence confirming its efficacy and safety in clinical practice.

On is essential for B-cell receptor signaling and the activation of downstream pathways that promote B-cell survival, proliferation, and autoantibody production.^23,24^ Inhibiting BTK can suppress aberrant B-cell activity and reduce the production of pathogenic autoantibodies that target platelet surface glycoproteins.^23,24^ By mitigating the autoimmune component of ITP, BTK inhibitors—such as rilzabrutinib—may not only decrease platelet destruction but also modify the underlying disease process.^23,24^ Unlike fostamatinib, rilzabrutinib and sovleplenib remain investigational agents currently under clinical development, with efficacy and safety data still limited to controlled trial settings.

In addition to these mechanistic advantages, both SYK and BTK inhibitors offer benefits as orally administered agents, which may contribute to better patient adherence and long-term treatment feasibility compared to parenteral therapies.^7^ The rapid onset of response observed in several trials also suggests that these agents can stabilize platelet counts effectively, potentially reducing the risk of bleeding in acutely symptomatic patients.^7^

Taken together, the dual capacity of SYK and BTK inhibitors to suppress immune-mediated platelet clearance and diminish autoantibody production underlies their clinical efficacy in improving platelet response in ITP.^7^ While these results support the integration of SYK and BTK inhibitors into the treatment paradigm for adult ITP—particularly in patients with inadequate responses or intolerance to conventional therapies, such as corticosteroids, TPO-RAs, and rituximab— direct head-to-head comparisons with established treatments are currently unavailable, limiting definitive conclusions about their optimal sequencing or relative effectiveness.^7^ Nevertheless, they may be considered as potential second- or third-line therapies, especially for patients who are refractory to or unable to tolerate existing immunosuppressive or thrombopoietic agents. Further studies exploring their role in combination regimens or as part of a stepwise treatment strategy could provide deeper insights into optimizing outcomes for patients with ITP.

Emerging next-generation BTK inhibitors such as orelabrutinib and zanubrutinib are attracting interest as targeted options for adult ITP because of their high BTK selectivity, favourable pharmacokinetics, and mechanistic potential to suppress pathogenic B-cell activity and FcγR-mediated macrophage platelet clearance. Early clinical evidence is encouraging: a phase II study of orelabrutinib reported promising efficacy and an acceptable safety profile in persistent or chronic primary ITP, supporting ongoing phase III evaluation.^25^ Similarly, zanubrutinib has shown activity in single-arm/phase II series and is being tested in randomized studies, including trials that combine zanubrutinib with eltrombopag.^26^ From a practical treatment-planning perspective, these BTK inhibitors could be considered as part of combination or sequencing strategies to broaden response mechanisms in refractory ITP. The rationale for combination is straightforward: pairing a BTK inhibitor (which reduces autoantibody generation and phagocytic platelet destruction) with a thrombopoietin-receptor agonist (which increases platelet production) may produce complementary and more durable hematologic control — early randomized data of zanubrutinib plus eltrombopag versus eltrombopag alone reported higher overall response rates at 12 months.^27^ Sequencing strategies are also plausible in routine practice: BTK inhibitors may be trialed after failure of first-line corticosteroids and either instead of, or following, TPO-RA or rituximab depending on individual patient comorbidity, prior response patterns, and treatment goals. Contemporary reviews therefore propose integrating BTK inhibitors into the second-/later-line algorithm for refractory disease while tailoring selection by patient preference, thrombotic/bleeding risks, and prior exposures.^7^

This study represents the first systematic review and meta-analysis to thoroughly evaluate and quantify the efficacy and safety of both SYK and BTK inhibitors in comparison to placebo for treating adult ITP. Unlike the earlier work by Ali MA et al.^6^, which was limited to a narrative systematic review without statistical aggregation of outcomes, our analysis provides a quantitative synthesis that allows for a clearer interpretation of treatment effects. Moreover, while the previous review^6^ included only four RCTs from three studies—one of which was a phase 1b trial—our current analysis incorporates five RCTs across four peer-reviewed publications, all of which were conducted at the phase 3 level. This strengthens the reliability of the findings and enhances their relevance to clinical decision-making. Accordingly, our study contributes more robust evidence that may help refine therapeutic strategies for adult ITP.

Despite offering valuable insights into the potential role of SYK and BTK inhibitors in the management of adult ITP, this meta-analysis is subject to several limitations that should be acknowledged. First, it included a relatively small number of RCTs, which restricts the statistical power of pooled estimates and limits the ability to conduct more granular subgroup analyses. This also precluded a robust assessment of publication bias, as such evaluations typically require at least 10 studies to yield meaningful conclusions. Second, most included trials reported outcomes over a relatively short follow-up period (24 weeks). As a result, the long-term efficacy, safety, and potential for sustained remission or relapse with SYK and BTK inhibitors remain uncertain. Third, all included studies compared SYK or BTK inhibitors to placebo; direct comparisons between different TKIs or between TKIs and established therapies (e.g., corticosteroids, TPO receptor agonists) were not available. This limits the ability to determine the relative positioning of these agents within the current treatment algorithm. Fourth, several outcomes in this meta-analysis showed notable degrees of heterogeneity, which may reflect differences in population characteristics, prior treatments, and dosing regimens. Such variability can influence the pooled estimates and limit the precision of the effect size. Although random-effect models were used to account for between-study variability, residual heterogeneity cannot be fully excluded and should be considered when interpreting the results. Fifth, although common AEs were consistently reported, the low incidence of rare or long-term toxicities, such as cardiovascular events or opportunistic infections, could not be thoroughly assessed. Larger sample sizes and extended observation periods are needed to capture these less frequent but clinically relevant events. Finally, most of the included trials were sponsored by pharmaceutical companies, which may introduce potential bias in trial design, data reporting, or interpretation of outcomes, despite adherence to rigorous methodological standards.

The findings of this systematic review and meta-analysis highlight the therapeutic potential of SYK and BTK inhibitors in the management of adult ITP. Compared to placebo, these agents were associated with significantly improved platelet response outcomes, including higher overall and durable response rates, more rapid achievement of platelet count thresholds, and decreased reliance on rescue interventions. Importantly, these clinical benefits were achieved without a corresponding increase in serious or high-grade AEs, suggesting a favorable risk–benefit profile. While these results support the integration of SYK and BTK inhibitors into the treatment paradigm for adult ITP—particularly in patients with inadequate responses or intolerance to conventional therapies—further high-quality, long-term RCTs are warranted to confirm these findings, assess sustained efficacy, and determine their comparative effectiveness against existing standard-of-care options.

### Authors’ Contribution

Conceptualization: Akhil D. Vatvani, Timotius I. Hariyanto, Alejandrina C. Ramirez, Nehad Shabarek; Methodology: Akhil D. Vatvani, Timotius I. Hariyanto, Alejandrina C. Ramirez; Formal analysis and investigation: Akhil D. Vatvani, Timotius I. Hariyanto, Alejandrina C. Ramirez, Nehad Shabarek; Writing - original draft preparation: Akhil D. Vatvani, Timotius I. Hariyanto, Alejandrina C. Ramirez; Writing - review and editing: Nehad Shabarek; Resources: Akhil D. Vatvani, Timotius I. Hariyanto, Alejandrina C. Ramirez, Nehad Shabarek; Supervision: Nehad Shabarek

### Competing of Interest – COPE

No competing interests were disclosed.

### Ethical Conduct Approval – Helsinki – IACUC

This is a systematic review and meta-analysis study. The Faculty of Medicine, Pelita Harapan University Research Ethics Committee has confirmed that no ethical approval is required.

### Informed Consent Statement

All authors and institutions have confirmed this manuscript for publication.

## Supplementary Material

Supplementary Table 1. Time to first platelet response outcome among the included studies Supplementary Table 1. Time to first platelet response outcome among the included studies

Supplementary Materials

## Data Availability

The authors confirm that the data supporting the findings of this study are available within the article [and/or] its supplementary materials.
