# Supplementary material for: Spleen and Bruton’s tyrosine kinase inhibitors for the management of adult immune thrombocytopenia: A systematic review and meta-analysis of randomized controlled trials
Source: Clin Hematol Int. 2025 Dec 24;7(4):76–91. doi: 10.46989/001c.154588 (PMC12742321; doi:10.46989/001c.154588)
Supplement: Supplementary Table 1. — Time to first platelet response outcome among the included studies [file chi_2025_7_4_154588_322822.pdf]

Supplementary Table 1. Time to first platelet response outcome among the included studies

Study name	Time to First Platelet Response	
	TK inhibitors	Placebo
Bussel J et al.[15] 2018	Median time was 15 days for overall responders and 15.5 days for stable responders	Not reported
Hu Y et al.[16] 2024	Median time = 8 (8 – 12) days	Median time = 30 (24 – 46) days
Kuter DJ et al.[17] 2025	Median time = 36 days	Never achieved

TK inhibitors = tyrosine kinase inhibitors

Supplementary Table 2. Bleeding events outcome among the included studies

Study name	Bleeding events	
	TK inhibitors	Placebo
Bussel J et al.[15] 2018	- Moderate or severe bleeding-related AEs occurred in 10/101 patients (9.9%)<br>- Bleeding-related SAEs occurred in 4/101 patients (3.9%)	- Moderate or severe bleeding-related AEs occurred in 8/49 (16%) patients<br>- Bleeding-related SAEs occurred in 5/49 patients (10%)
Hu Y et al.[16] 2024	- Total bleeding events reduced significantly from 87/126 patients (69%) at baseline to 23/86 patients (27%) at week 24	- Total bleeding events reduced significantly from 33/62 patients (53%) at baseline to 1/8 patients (13%) at week 24
Kuter DJ et al.[17] 2025	- Grade ≥2 bleeding events due to any cause were reported in 4% of patients	- Grade ≥2 bleeding events due to any cause were reported in 12% of patients
Kuwana M et al.[18] 2023	- Bleeding events occurred in 3/22 patients (14%)	- Bleeding events occurred in 2/12 patients (17%)

AEs = adverse events; SAEs = serious adverse events; TK inhibitors = tyrosine kinase inhibitors

Study name	Statistics with study removed				
	Point	Lower limit	Upper limit	Z-Value	p-Value
Bussel J et al. 2018 (grade 1-2)	3.020	1.540	5.924	3.215	0.001
Kuter DJ et al. 2025 (grade 1-2)	1.057	0.649	1.722	0.225	0.822
Kuwana M et al. 2022 (grade 1-2)	1.577	0.726	3.423	1.152	0.249
Bussel J et al. 2018 (grade >3)	1.855	0.757	4.541	1.352	0.176
Kuter DJ et al. 2025 (grade >3)	1.920	0.809	4.555	1.479	0.139
Kuwana M et al. 2022 (grade >3)	1.833	0.748	4.493	1.325	0.185
Pooled	1.793	0.812	3.955	1.446	0.148
Prediction Interval	1.793	0.263	12.238	0.000	0.000

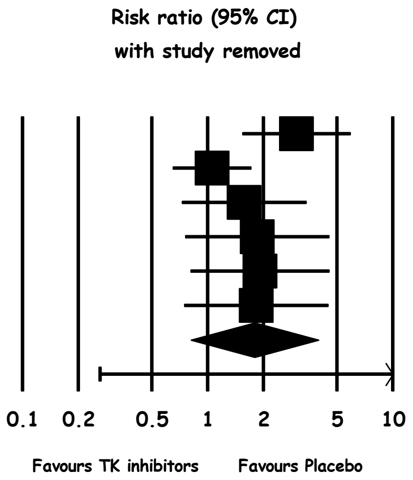

Supplementary Figure 1. Sensitivity analysis by leave-one-out method for the overall diarrhea incidence outcome.

Study name	Statistics with study removed				
	Point	Lower limit	Upper limit	Z-Value	p-Value
Bussel J et al. 2018 (grade 1-2)	3.433	1.673	7.042	3.364	0.001
Kuter DJ et al. 2025 (grade 1-2)	2.006	0.249	16.180	0.654	0.513
Kuwana M et al. 2022 (grade 1-2)	1.705	0.534	5.447	0.901	0.368
Pooled	2.110	0.694	6.419	1.316	0.188
Prediction Interval	2.110	0.000	512012.311	0.000	0.000

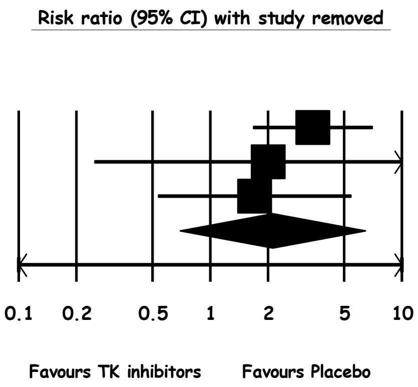

Supplementary Figure 2. Sensitivity analysis by leave-one-out method for the grade 1-2 diarrhea outcome.

Study name	Statistics with study removed				
	Point	Lower limit	Upper limit	Z-Value	p-Value
Bussel J et al. 2018 (grade 1-2)	0.806	0.144	4.515	-0.245	0.807
Hu Y et al. 2024 (grade 1-2)	0.603	0.198	1.840	-0.888	0.375
Kuter DJ et al. 2025 (grade 1-2)	0.873	0.198	3.850	-0.179	0.858
Kuwana M et al. 2022 (grade 1-2)	1.388	0.594	3.240	0.757	0.449
Pooled	0.943	0.316	2.819	-0.104	0.917
Prediction Interval	0.943	0.011	82.218	0.000	0.000

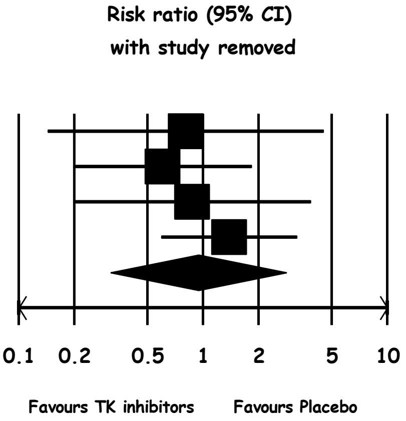

Supplementary Figure 3. Sensitivity analysis by leave-one-out method for the grade 1-2 upper respiratory infection outcome.
